# Supplementary material for: Statistical estimates of multiple transcription factors binding in the model plant genomes based on ChIP-seq data
Source: J Integr Bioinform. 2021 Dec 21;19(1):20200036. doi: 10.1515/jib-2020-0036 (PMC9069649; doi:10.1515/jib-2020-0036)
Supplement: Supplementary file 1 — Supplementary Material Details [file j_jib-2020-0036_suppl.docx]

Statistical estimates of multiple transcription factors binding in the model plant genomes based on ChIP-seq data

Dergilev A.I.^1,2^, Orlova N.G.^3,4^, Dobrovolskaya O.B.^2,5^, Orlov Y.L.^1,2,5,6^

^1^ Novosibirsk State University, 630090 Novosibirsk, Russia

^2^ Institute of Cytology and Genetics, Siberian Branch of the Russian Academy of Sciences, 630090 Novosibirsk, Russia

^3^ Financial University under the Government of the Russian Federation, 125993 Moscow, Russia

^4^ Moscow State Technical University of Civil Aviation, 125993 Moscow, Russia

^5^ Agrarian and Technological Institute, Peoples’ Friendship University of Russia, 117198 Moscow, Russia

^6^ The Digital Health Institute, I.M.Sechenov First Moscow State Medical University (Sechenov University), 119991 Moscow, Russia

# Abstract

The development of high-throughput genomic sequencing coupled with chromatin immunoprecipitation technologies allows studying the binding sites of the protein transcription factors (TF) in the genome scale. The growth of data volume on the experimentally determined binding sites raises qualitatively new problems for the analysis of gene expression regulation, prediction of transcription factors target genes, and regulatory gene networks reconstruction. Genome regulation remains an insufficiently studied though plants have complex molecular regulatory mechanisms of gene expression and response to environmental stresses. It is important to develop new software tools for the analysis of the TF binding sites location and their clustering in the plant genomes, visualization, and the following statistical estimates. This study presents application of the analysis of multiple TF binding profiles in three evolutionarily distant model plant organisms. The construction and analysis of non-random ChIP-seq binding clusters of the different TFs in mammalian embryonic stem cells were discussed earlier using similar bioinformatics approaches. Such clusters of TF binding sites may indicate the gene regulatory regions, enhancers and gene transcription regulatory hubs. It can be used for analysis of the gene promoters as well as a background for transcription networks reconstruction. We discuss the statistical estimates of the TF binding sites clusters in the model plant genomes. The distributions of the number of different TFs per binding cluster follow same power law distribution for all the genomes studied. The binding clusters in *Arabidopsis thaliana* genome were discussed here in detail.

**Keywords:** transcription factor binding sites; transcription regulation; gene expression; ChIP-seq; regulatory gene networks; plant genomes

# Introduction

The regulation of gene transcription plays a key role in cell functioning (Ignatieva et al., 2015). The study of transcription regulation based on the transcription factor (TF) binding data is one of the most developed bioinformatics fields. On-going acquisition of the data on gene expression and binding sites obtained by sequencing techniques such as ChIP-seq, BS-seq, DNaseI-seq, ATAC-seq, NOMe-seq (Orlov et al., 2012; Dergilev et al., 2016; Chen et al., 2018) demands development of data integration. Qualitatively new problems arise for combinatorial gene expression regulation based on multiple TFs binding. Does multiple TF binding in a gene region determine organism development program, or it is just random effects not related to gene function (Kazemian et al., 2013)? Statistical estimates of multiple TFs binding may highlight fundamental gene expression features common for any eukaryotic genome (Ramsey, 2016). Linking the gene promoter regions and high-throughput TF binding data we may reconstruct regulatory gene networks in the genome scale (Ignatieva et al., 2017). The location of a TF binding site in genomic sequence may be remote from the gene transcription start, thus preventing reliable TF target gene prediction. Despite the development of data sophisticated analysis techniques, the prediction of the regulatory regions of transcription, based only on the nucleotide sequence or single binding events remains a complex problem.

New problems arise in the analysis of the joint location (co-localization) of the binding sites of different protein transcription factors in the same loci (Tsukanov et al., 2018; Dergilev et al., 2016). There are cooperative regulatory effects with the simultaneous binding of various transcription factors in protein complexes to DNA (Orlov et al., 2014; Narasimhan et al., 2015; Khoueiry et al., 2017). It is necessary to develop the tools and methods of the sequencing data processing, evaluation of binding effects of transcription factors (Dergilev et al, 2017; Kulakova et al, 2015; Kulakova et al, 2017; Khoueiry et al, 2017).

We used experimental ChIP-seq data to model TF binding as the alternative to sequence-based prediction. Co-location of binding sites of two or more different factors in the promoter region of the same target gene may define an element of a regulatory gene network. Several protein transcription factors may bind to the same gene promoter region that corresponds to network interactions “TFs - gene”. One TF may have several gene targets that corresponds to “TF - genes” interactions. Protein TF may bind to the promoter of its own gene, thus forming a regulatory contour in gene network. Thus, a regulatory gene network can be reconstructed from a set of the binding sites of several different TFs. Such a regulatory network might be conserved between species. The examples of the mutual regulation of several pluripotency factors in embryonic stem cells in mouse were shown (Chen et al., 2008). The dynamic models of regulatory gene networks have also been developed for the pluripotency factors binding to the same regulatory regions (Akberdin et al., 2018). Possible overlapping of binding sites on the same nucleotide sequence of multiple TF binding clusters, and mutual location of the binding sites may follow certain regularities and rules to be found by statistics of DNA oligonucleotides (Vityaev et al., 2001; Vityaev et al., 2002).

A physical explanation of the observed binding patterns on DNA may be due to the protein-protein interactions between the TFs bound as multimer complex. Protein transcription factors can directly contact each other, simultaneously bind to DNA in neighboring regions, or compete for binding to the same DNA sequence in time (Khoueiry et al., 2017). ChIP-seq data generally do not provide an experimental answer to the question: “Do the TFs bind the same genome locus simultaneously or the binding events differ in time?” (Chen et al., 2008; Khoueiry et al., 2017). Thus, we can only estimate frequency of the interactions, but not prove cooperative binding of the proteins to the same DNA site.

The binding regulatory region could be assessed taking into Account Topologically Associated Domains (TAD) in the chromosomes, which could be revealed using sequencing technologies based on chromosome conformation capture (3C, with genome-wide variants Hi-C and ChIA-PET) (Fullwood et al., 2009; Li et al., 2012; Kulakova et al., 2015; Orlov et al. 2017). Clusters of the binding sites on the chromosomes mark topological domains studied mainly in mammalian genomes (Li et al., 2012). Chromosome conformation data (Hi-C) were obtained initially for Arabidopsis, and recently for other crop plants models. TADs are absent in Arabidopsis but they are found in more complex plant crop genomes including rice (Grob, 2020).

In general, the problem of joint gene expression regulation by a set of TFs has not been sufficiently studied (Ibarra et al., 2020; Tsukanov et al., 2018). For plant genomes, such studies are presented in separate databases (Muhammad et al., 2019; Orlov et al. 2019; Chow et al., 2019; Hao et al., 2019; Zakhartsev et al., 2016). Despite different models of TF-DNA binding, statistical estimates of the distribution of the number of different TF in binding clusters were not discussed. We applied earlier developed computer scripts (Dergilev et al., 2016) to analyze ChIP-seq data, calculate clusters and visualize them in the form of heat maps in plants.

We used ChIP-seq data of three plants, including *Arabidopsis thaliana, Physcomitrella patens,* and *Chlamydomonas reinhardtii* to model TF binding clusters in their genomes and estimate the distribution of the TF in the clusters found.

# Materials and methods

## Current version of TFBS Tool Analysis Portable

A set of scripts in the Python language has been developed earlier for computer analysis of clusters of binding sites for transcription factors (see Figure 1) with a graphical shell implemented in the Qt 5 environment (Dergilev et al., 2016). We compiled it to the cross-platform software tool ‘TFBS Tools Analysis’.

For the full usage of TFBS Analysis Portable, additional software is required: an installed Python interpreter of version 3.5 or higher (https://www.python.org/downloads/windows/), programming environment R version 3.4.1 or higher (https://www.r-project.org/), Git Bash for Windows (https://gitforwindows.org/).

**A**


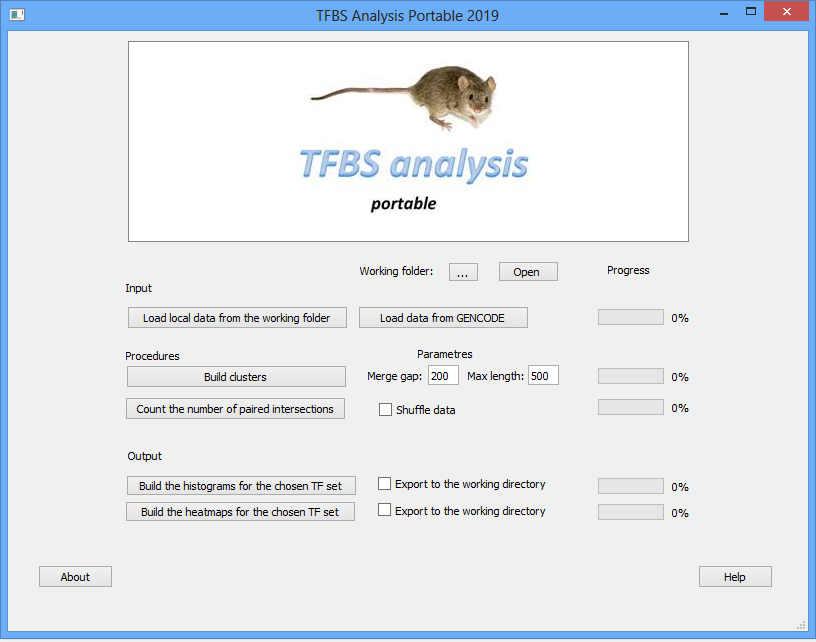


**B**

**Fig. 1:** TFBS Analysis Portable. A ‑ toolbox main window; B - block-scheme of the data processing.

TFBS Analysis Portable allows conveniently convert and analyze the TF binding sites data in genome scale, namely:

1. Load data (TF binding sites coordinates in a simplified BED format)

2. Load the chromosome coordinates in GFF 3 data format (option including subsequent data conversion using an additional script).

3. Build coordinates of the clusters of TFs binding sites iteratively based on coordinates of single sites (adjusting the parameters of the gap between the coordinates of the sites and the maximum cluster region size, in nucleotides).

4. Calculate pairwise intersections of the TFs binding loci in the genome.

5. Construct the statistical histograms of the cluster occurrence for each TF separately and in the groups.

6. Build heat maps of TF co-binding in the given genome.

The program did not filter the initial data on the quality of DNA reads, on the basis of which the binding sites of transcription factors in the genome were determined. However, such data pre-processing is necessary (Vasiliev et al., 2017; Abnizova et al., 2017; Naumenko et al., 2018) and can be included in the general pipeline of analysis and subsequent refinement of site clusters. The detection of binding sites from DNA reads in ChIP-seq experiments provides a set of genome loci for a given TF. ChIP-seq experiments give several sets of TF binding sites in genome (chromosome) coordinates. Comparing the binding coordinates we construct a set of common loci, occupied by two or more TF - i.e. a binding cluster. The width of such clusters in genome is limited by the distance parameter (normally less than 500 nucleotides). The size 500 nt refers to the standard promoter size. It corresponds to the distance where proteins bound to DNA can physically interact to each other. This parameter may vary in the program runs. We adjusted it by the calculation of distance between peaks in ChIP-seq genomes profiles to 200 nt. The number of different TF in the binding cluster is not limited (up to all available TF data set in the genome under study).

## The data and algorithms

We download available ChIP-seq data from UCSC Genome Browser (http://genome.ucsc.edu/) and the plant genome databases (Tian et al, 2020). The TFBS Analysis Portable tool has flexibility in relation to various genomes differing by genome size and number of chromosomes. Currently, this code is available upon request to the authors. The data in the form of BED files were downloaded from the databases: NCBI (https:www.ncbi.nlm.nih.gov/geo/query/acc.cgi?acc=GSE24568) (Wheeler et al., 2015), PlantTFDB (http:planttfdb.cbi.pku.edu.cn, see also http://ucsc.gao-lab.org/) (Jin et al, 2014), PlantRegMap (http:plantregmap.cbi.pku.edu.cn) (Tian et al, 2020).

In total we have downloaded the ChIP-seq peaks for 28 transcription factors for *Arabidopsis thaliana* (flowering plants) (Fridborg et al., 1999), 18 factors for *Physcomitrella patens* (moss) and 8 factors for *Chlamydomonas reinhardtii* (green algae, plant general type) (Wheeler et al., 2015; Jin et al, 2014; Tian et al, 2020).

These plants present taxons from a simple unicellular alga to a more complex flowering plant. The evolutionary relationship between these plants can be seen on the phylogenetic tree shown in Figure 2 (Soltis et al., 2003).


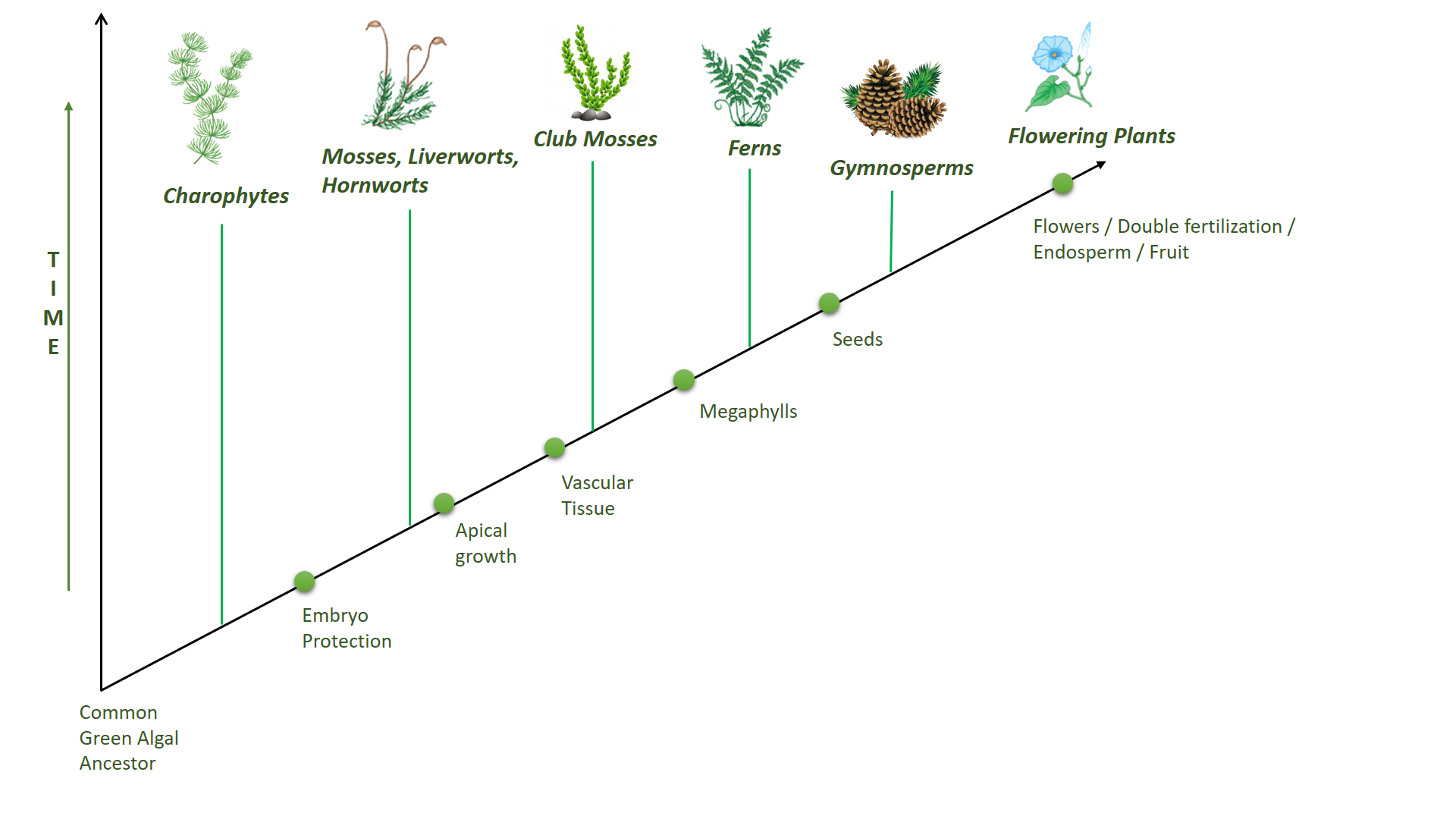


**Fig. 2:** A schematic representation of a phylogenetic tree representing the process of plant evolution over time. The order of genomes studied is from *mosses and algae (Chlamydomonas reinhardtii,* and *Physcomitrella patens)* to more complex flowering plants (*Arabidopsis thaliana).*

Note the detailed databases on genome-wide TF binding developed earlier - TRRD, GTRD, and HOCOMOCO (Ignatieva et al., 2017; Kolmykov et al., 2021; Kulakovskiy et al., 2018). However, data on TF binding in plants are rather scattered (Fridborg et al., 1999; Chow et al., 2019; Hao et al., 2019; Gorshkov et al., 2019). For comparison with predicted binding sites in Arabidopsis we used AthaMap resource (Hehl et al., 2016) and JASPAR (Wasserman and Sandelin, 2004).We plan to integrate novel available data on TF binding in plants (Di Stefano et al., 2021).

Transcription factor binding sites tend to be found together, especially in gene promoter regions. See example for *A.thaliana* gene AT1G01040 from plant genome browser (Figure 3) (<http://ucsc.gao-lab.org/>). Conserved TF binding sites overall each other in chromosome coordinates.


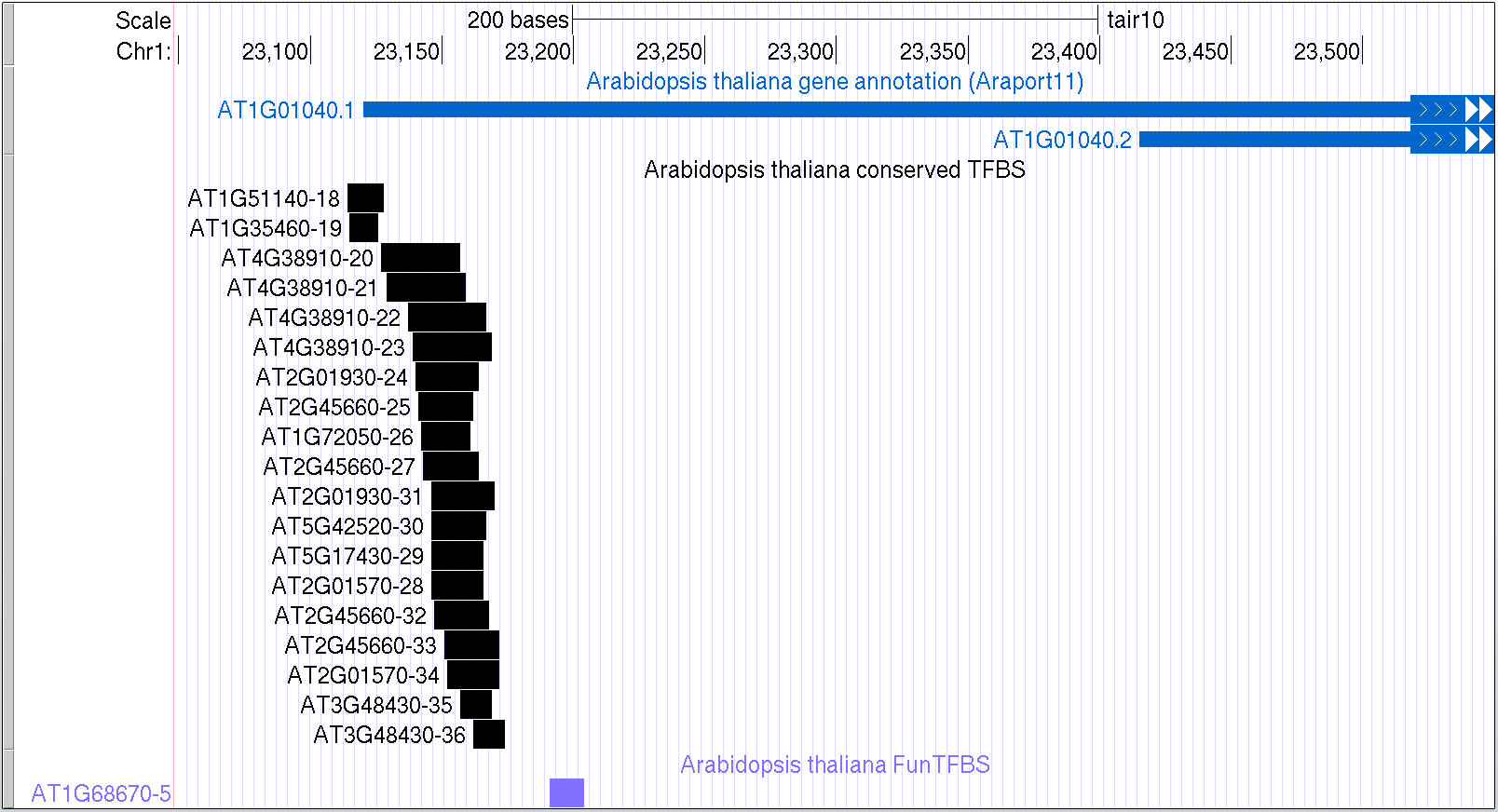


**Fig. 3:** The example of multiple transcription factors binding in the *A.thaliana* genome in promoter region of AT1G01040 (DCL1) gene (The plant genome browser, <http://ucsc.gao-lab.org/>).

To construct the clusters, we have iteratively compared the coordinates of the binding sites for each TF. The ChIP-seq peaks (summit position in the given ChIP-seq profile) were counted as the binding site positions. Thus, we used experimental data instead of sequence-based prediction. However, the tool can take as input data any set of genome coordinates, both experimental and predicted. The distances between the sites (peaks) were compared, counting for the new cluster if the distance was less than 200 nt. Figure 4 shows scheme of cluster counting by TF binding coordinates.


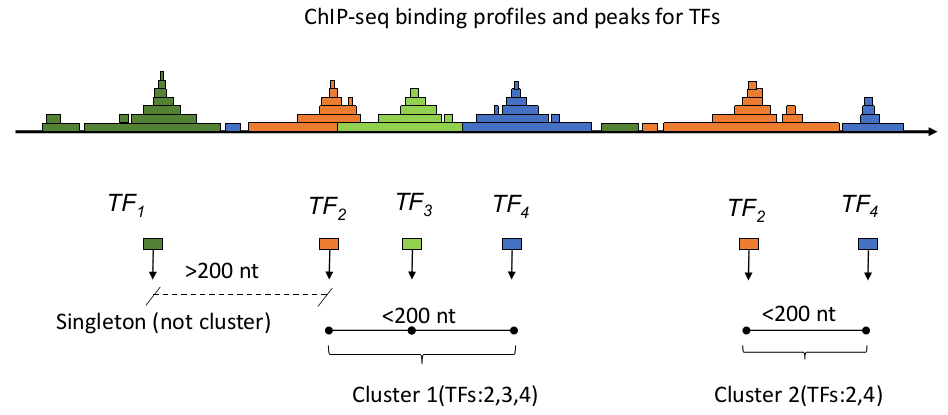


**Fig. 4:** Example of the processing of ChIP-seq profiles (peaks) for the TFs binding to count binding clusters in genome coordinates.

The parameter for the clusters construction is 200 nt gap between the experimentally determined positions of the binding sites in genome coordinates. If taking a longer distance (500 nt) many not related TF sites would be counted in clusters. If considering only neighboring TF binding sites, with a distance less than 20 nt in between, the maximum cluster size is reduced while maintaining the shape of the distribution of the number of sites (data not shown). We had followed the algorithm of cluster construction described first in (Chen et al., 2008) and later extended in (Tsukanov et l., 2018).

## Functional features of the studied data

We used all available ChIP-seq data for TFs binding in the chosen model plants. These TFs are important for plant development and were previously studied (Chow et al., 2019). The following sets of TFs ChIP-seq data were counted:

*Arabidopsis thaliana*: AP2, ARF, B3, BBR, BPC, bZIP, C2H2, CH3, CAMTA, CPP, DOF, E2F / DP, ERF, FAR1, G2-like, GATA, GRAS, HSF, HD-ZIP, LBD, LFY, MADS, MYB, NAC, SRS, TCP, WRKY, YABBY.

*Physcomitrella patens*: ARF, bZIP, C3H, CAMTA, CPP, WOX, Dof, E2F / DP, ERF, G2, GATA, HD- ZIP, LBD, LFY, MYB, NAC, TCP, WRKY.

*Chlamydomonas reinhardtii*: C2H2, C3H, CPP, Dof, ERF, G2-like, GATA, MYB.

Consider functions of these transcription factors in brief. AP2 / ERF regulate plant development and response to various types of biotic and environmental stress. ARF is a key regulator of virtually all aspects of plant growth and development, from embryogenesis to aging.

In plants, transcription factors of the leucine zipper motif (bZIP) regulate the processes of protection against pathogens, transmission of light and stress signals, seed maturation and flower development. C2H2 is a sequence-specific DNA binding protein regulating transcription. The GATA factors form a class of transcription regulators present in mushrooms, animals and plants which generally recognize a consensus sequence WGATAR (Lowry and Atchley, 2000).

The plant-specific transcription factor LEAFY (LFY) plays a central, evolutionarily conserved role in the transition from vegetative growth to flowering (Winter et al., 2011). MADS are DNA-binding proteins that play an essential role in a variety of biological processes in the development and flower plants (Lai et. al, 2019). TCP are plant-specific transcription factors that play an important regulatory role in plant growth (Zheng et al., 2018). WRKY are one of the largest families of transcriptional regulators found exclusively in plants). They perform a variety of biological functions in relation to plant resistance to diseases, response to abiotic stress and processes controlled by hormones.

The main function of the YABBY genes is to determine the fate of abaxial cells in the lateral organs of Arabidopsis (Villanueva et al. 1999; Cong et al. 2008). In particular, YABBY-regulated WRKYs play an important role in the regulation of plant stress responses, both as activators and repressors. Overall, the TFs used in the study were compiled by ChIP-seq data availability in the genomes, not restricting to the function.

# Results and discussion

Using coordinates of ChIP-seq binding peaks we have iteratively compared the binding sites coordinates and constructed the coordinates of the clusters for every genome. The statistical histograms have been built for all the plants studied. Figure 5 shows the number of clusters depending on the cluster size (the number of sites it contains)


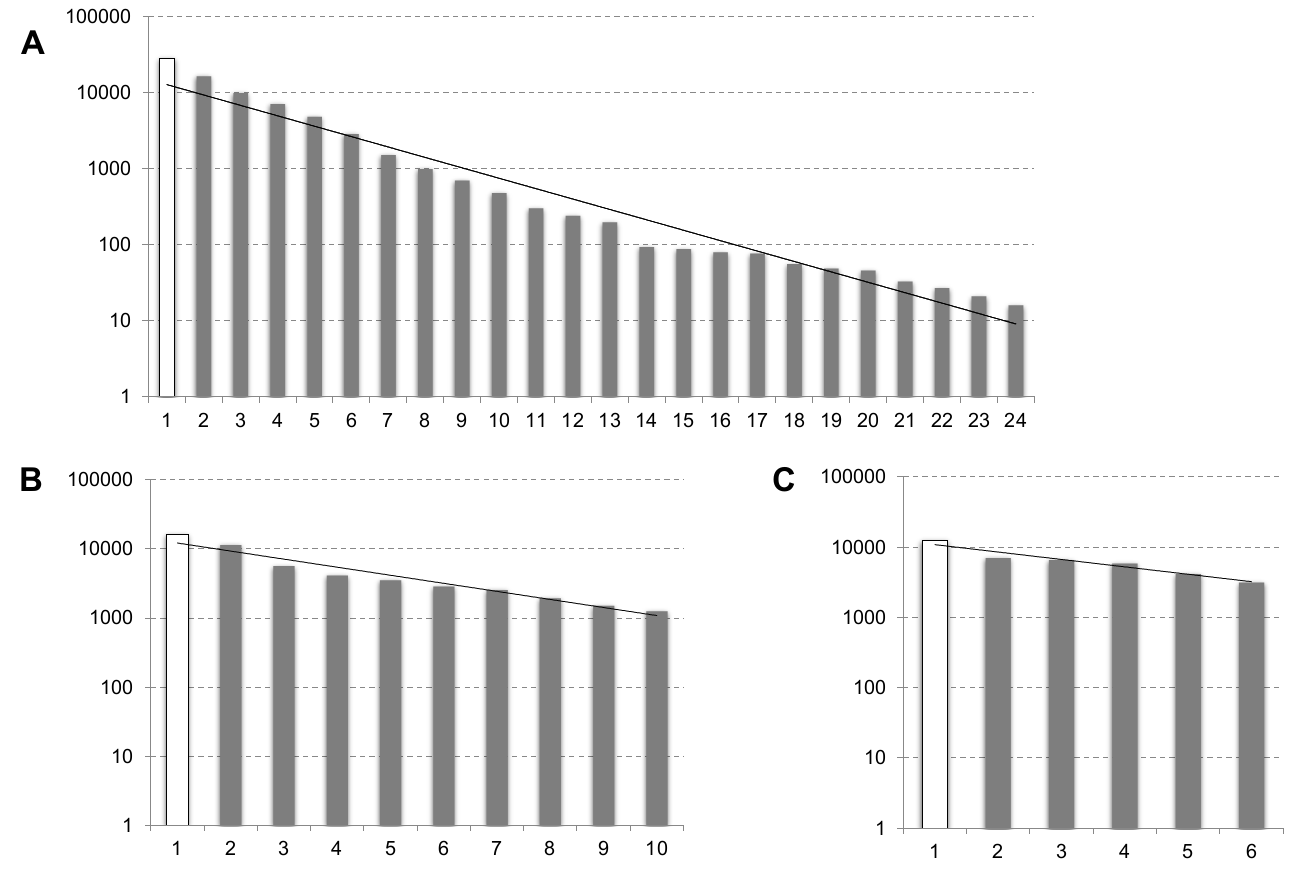


**Fig. 5:** Dependence of the number of clusters of binding sites on cluster size.
A - *Arabidopsis thaliana*, B - *Physcomitrella patens*, C - *Chlamydomonas reinhardtii*. Axis X shows the number of sites in the cluster, axis Y - the number of such clusters. Single binding sites (size 1, singletons) are shown by the hollow column. The values are displayed on semi-logarithmic scale.

The probability of clustering several binding sites decreases by Power law distribution. One can see it from the observed histograms in log-scale (with some variability). Clusters of sites with a size of 4 and higher in the genome can no longer be explained by random causes, as it was shown previously earlier theoretically and using computer simulations (Chen et al., 2008). The number of different TF in a binding cluster generally obeys the distribution of the probability of obtaining group of elements of a given size (Kuznetsov et al., 2007; Wang et al., 2017). However, the observed distribution of the number of different TF per cluster differs from the expected by chance: it follows power-law distribution with longer right-tail (clusters of very large size not expected by chance).

The figure shows the presence of large clusters (two, three or more different TFs). There are up to several hundred binding clusters, for each of the considered plants.

It is interesting to note the existence of very complex clusters containing binding sites for almost all transcription factors available (ten or more TFs). For *Arabidopsis thaliana*, the maximum cluster size is 24. For *Physcomitrella patens* – the maximal size is 10. For *Chlamydomonas Reinhardtii* - 18.

Thus, such regions could act as super-enhancers. The term super enhancer was introduced in the literature for gene regulatory regions mediating expression of many genes in different tissues first for stem cells (Whyte et al., 2013). Super-enhancers are large clusters of transcriptional enhancers that are co-occupied by multiple lineage-specific transcription factors driving expression of genes that define cell identity. For plants such enhancer may drive development programs, switch plant cell response to external factors. If we consider structure of gene regulatory network the gene having such large TF binding cluster in its promoter region will be seen as a network hub with multiple links.

Statistical histograms were constructed showing the individual occurrence of each factor for a given set of plants (Figure 6).

**Fig. 6:** The occurrence of each transcription factor in the clusters (in the range [0;1]). A - *Arabidopsis thaliana*, B - *Physcomitrella patens*, C - *Chlamydomonas reinhardtii*. The name of the factor is on the horizontal axis, and its occurrence is shown on the vertical axis.

It is seen from the figures that the number of clusters decreases exponentially with the size of the cluster. This dependence approximately follows Power law distribution. It is theoretically expected. At the same time, there is no genomic region containing simultaneously the sites of all factors for each plant which indicates their functional differences.

Let’s consider the functions of some factors found in the binding clusters.

It can be seen that the LFY factor, which is responsible for the growth of flowers in plants, tends to occur in the Arabidopsis genome most often among all the others, while the SRS group factors are least expressed (Fridborg et al., 1999).

In the *Physcomitrella patens* genome, LFY is again quantitatively in the first place, while G2, which participates in the process of the cell cycle and influences cell differentiation, occurs less frequently than others.

The CPP family of factors in *Chlamydomonas reinhardtii* (Riechmann et al., 2000) tends to occur more often, and the factors of the group G2 like are less common.

For each pair of TFs we count number of binding clusters and put this integer number to a matrix. It is a symmetrical matrix. At the next stage for each pair of transcription factors we calculated the Pearson linear correlation coefficient by the rows (vectors of the integer numbers) from this matrix (Dergilev et al., 2016). Each cell of such a symmetric matrix contains the number of binding sites (overlapping in a given genomic range up to 200 nt) of two factors. It is possible to use rank correlation coefficients as well as linear coefficients to follow the work by Chen et al. (Chen et al., 2008). Thus, the matrix of integer numbers was translated into a correlation matrix (float numbers in [0;1] interval). The correlation coefficient gives normalization of the values for the sets of transcription factors sites of different sizes. Such a matrix provides a measure of association, between the binding sites for the pairs of transcription factors. The higher the correlation coefficient is, the closer the location of the binding sites of the transcription factors in the genome. The matrix can be represented in the form of a heat map, where the color intensity shows the correlation (the correlation coefficient).

Using this measure in the R programming environment (https://www.r-project.org/) the sites of the transcription factors were clustered with the following visualization. Figure 7 presents the heatmaps that highlight the intensity of joint binding of transcription factors in the genomes studied.


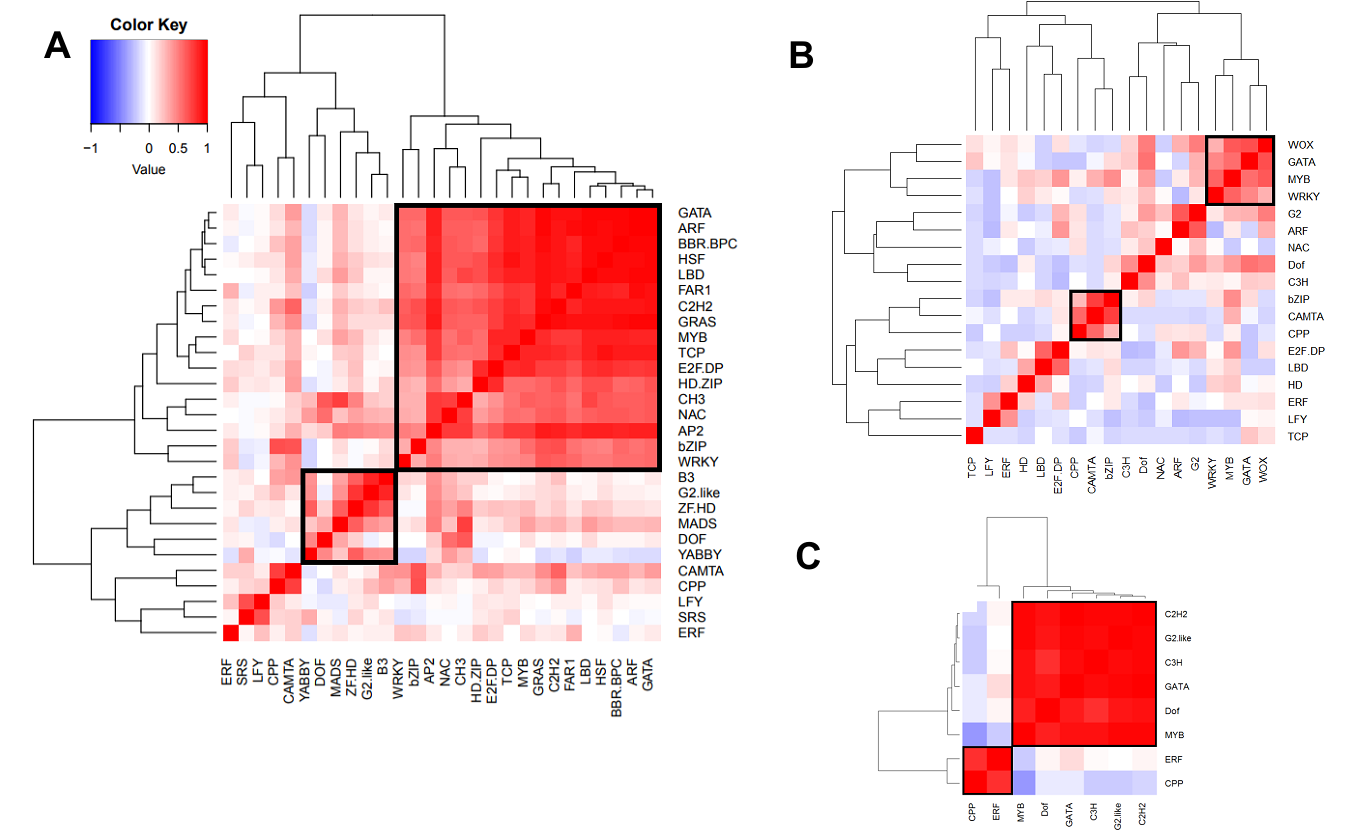


**Fig. 7:** Heat map of joint localization of TFs in the plants studied. Red is a more frequent occurrence. The squares highlight the groups of factors that tend to occur together most frequently.

Figure 7 shows that the same factors GATA and MYB are in the largest cluster in all the genomes.

In the investigated set of 28 factors of the plant *Arabidopsis thaliana,* the heatmaps allow delineating two major groups of TFs: {GATA, ARF, BBR-BPC, HSF, LBD, FAR1, C2H2, GRAS, MYB, TCP, E2F/DP, HD-ZIP, CH3, NAC, AP2, bZIP, WRKY} and {B3, G2-like, ZF-HD, MADS, DOF, YABBY}, or, in other words, relating to the GATA and related G2-like transcription factors.

It is also interesting to note that the LFY factor tends to occur most often among all the others, but it correlates weakly with other factors (except with SRS). Among the selected 28 factors, GATA, ARF, BBR-BPC and HSF tend to occur together more often.

In the studied set of 18 factors of the *Physcomitrella patens* plant, the situation looks different: the factors are rather weakly correlated with each other, so it is difficult to distinguish large groups of co-occurring factors. Moreover, WOX (Costanzo et al., 2014) and GATA (Tong et al., 2005) occur together more often.

It is known that the interaction between GATA and the C/EBP family of transcription factors (which includes the bZIP factor considered in the work and some others) is critical for the GATA-mediated suppression of adipocyte differentiation (Tong et al., 2005). It is interesting to note that C/EBP interacts with the CCAAT box motif in several gene promoters. They are characterized by a highly conserved basic leucine zipper (bZIP) domain at the C-terminus.

In the set of 8 factors of the *Chlamydomonas reinhardtii* plant, two groups are clearly distinguished: { C2H2, C3H, CPP, Dof, G 2- like, GATA, MYB } and { CPP, ERF }.

In general, it can be noted that the evolutionarily more ancient factors GATA and MYB are presented in the clusters of sites in all studied plant species. The flowering plant-specific LFY factor is presented in binding clusters in Arabidopsis. Clustering of sites in the genome for various transcription factors can be affected by the context features of the nucleotide sequence (nucleotide composition and motifs) of a regulatory region, by the structure of the protein that determines the type of binding to DNA, and has an evolutionary constraint (Vityaev et al., 2001). At the same time, epigenetic modifications, and the binding of TF to DNA in a specific tissue and plant organ should be considered. These sequencing data are currently insufficient for such assessments (Chow et al., 2019).

The general revealed regularity of the existence of clusters of binding sites in eukaryotic genomes, shown for the mouse and human genomes (Chen et al., 2008; Dergilev et al., 2016), is confirmed for plants. The general pattern in the distribution of site cluster sizes corresponds to the number of links in models of complex biological networks (Jeong et al., 2000), including networks of protein-protein interactions (Rolland et al., 2014). Clusters of TF binding sites can be found using the proposed statistical estimates. Visualization of such cluster using heat maps is a convenient bioinformatics method (Kulakova et al., 2017).

# Conclusion

The developed computer approach for clustering of variable sets of sites can be applied to a wide range of problems of assessing functional elements in the genome. In addition to TF binding the sites may include areas of low text complexity, tandem repeats, and CpG islands (Babenko et al., 2018). Analysis of functional clusters makes it possible to statistically describe enhancers, annotate genomes, in cases where experimental information is not available. Integration of experimental genomic information, Big Data, in general, represents an important problem in bioinformatics, requiring the integration of existing software tools and approaches (Chen et al., 2017; Orlov et al., 2020; Orlov et al., 2021). The presented statistical analysis of clusters of binding sites in plant genomes helps in solving the problem, developing approaches to the study of the evolutionary origin of enhancers and gene networks (He et al., 2012; Dobrovolskaya et al., 2015). The exact positioning of sites in the clusters is affected by both nucleosome packing (Goh et al., 2010) and spatial restrictions on the sequence length in topologically associated domain, where protein transcription factors interact with DNA (Li et al., 2012), thus arranging the binding sites by complex rules yet to be revealed.

For the considered plant genomes, analysis of an expanded set of binding sites for transcription factors confirmed a broad joint clustering of binding sites for transcription factors of the GATA family, that is, the factors characterized by their ability to bind to the GATA DNA sequence. Contrary to expectations, the LFY factor weakly correlates with the other factors studied.

In general, the proposed statistical estimates allow identifying non-random clusters of TF binding sites in plant genomes. The distribution of site clusters by size shows the general patterns of the clusters site formation in eukaryotic genomes. Such gene regulatory regions in the plant genome should be further investigated by experimental methods to identify cooperative interactions, to determine the functional role of the TF binding clusters, including response of the plant to stress conditions (Doroshkov et al., 2019).

**Acknowledgements**

The authors are grateful to A.E. Dresvyannikova, R.O. Babenko, V.A. Kononov for scientific discussion and assistance in the work. The publication has been prepared with the support of the RUDN University Strategic Academic Leadership Program (Recipients: YO, OD).

# References

- - - 1. Abnizova I., te Boekhorst R., Orlov Y. Computational Errors and Biases of Short Read Next Generation Sequencing. J Proteomics Bioinform. 2017; 10: 1-17. doi: 10.4172/jpb.1000420
      2. Akberdin I.R., Omelyanchuk N.A., Fadeev S.I., Leskova N.E., Oschepkova E.A., Kazantsev F.V., Matushkin Y.G., Afonnikov D.A., Kolchanov N.A. Pluripotency gene network dynamics: System views from parametric analysis. PLoS One. 2018;13(3):e0194464. doi: 10.1371/journal.pone.0194464
      3. Babenko V.N., Bogomolov A.G., Babenko R.O., Galieva E.R., Orlov Y.L. CpG islands’ clustering uncovers early development genes in the human genome. Computer Science and Information Systems, 2018; 15(2): 473-485 (2018) doi: 10.2298/CSIS170523004B
      4. Bouché N., Scharlat A., Snedden W., Bouchez D., Fromm H. A novel family of calmodulin-binding transcription activators in multicellular organisms. J Biol Chem. 2002;277(24):21851‐21861. doi:10.1074/jbc.M200268200
      5. Chen M., Harrison A., Shanahan H., Orlov Y. Biological Big Bytes: Integrative Analysis of Large Biological Datasets. J Integr Bioinform. 2017; 14(3). doi: 10.1515/jib-2017-0052
      6. Chen X., Bhadauria V., Ma B. ChIP-Seq: A Powerful Tool for Studying Protein-DNA Interactions in Plants. Curr Issues Mol Biol. 2018; 27:171-180. doi: 10.21775/cimb.027.171.
      7. Chen X., Xu H., Yuan P., Fang F., Huss M., Vega V.B., Wong E., Orlov Y.L., Zhang W., Jiang J., Loh Y.H., Yeo H.C., Yeo Z.X., Narang V., Govindarajan K.R., Leong B., Shahab A., Ruan Y., Bourque G., Sung W.K., Clarke N.D., Wei C.L., Ng H.H. Integration of external signaling pathways with the core transcriptional network in embryonic stem cells. Cell. 2008; 133(6):1106-17. doi: 10.1016/j.cell.2008.04.043
      8. Chow C.N., Lee T.Y., Hung Y.C., Li G.Z., Tseng K.C., Liu Y.H., Kuo P.L., Zheng H.Q., Chang W.C. PlantPAN3.0: a new and updated resource for reconstructing transcriptional regulatory networks from ChIP-seq experiments in plants. Nucleic Acids Res. 2019; 47(D1):D1155-D1163. doi: 10.1093/nar/gky1081.
      9. Costanzo E., Trehin C., Vandenbussche M. The role of WOX genes in flower development. Ann Bot. 2014; 114(7):1545-53. doi: 10.1093/aob/mcu123.
      10. Dergilev A.I., Spitsina A.M., Chadaeva I.V., Svichkarev A.V., Naumenko F.M., Kulakova E.V., Vityaev E.E., Chen M., Orlov Y.L. Computer analysis of colocalization of the TFs’ binding sites in the genome according to the ChIP-seq data. Russian Journal of Genetics: Applied Research. 2017; 7(5):513-522. doi: 10.18699/VJ16.194
      11. Di Stefano M., Nützmann H.W., Marti-Renom M.A., Jost D. Polymer modelling unveils the roles of heterochromatin and nucleolar organizing regions in shaping 3D genome organization in Arabidopsis thaliana. Nucleic Acids Res. 2021; 49(4):1840-1858. doi:10.1093/nar/gkaa1275
      12. Dobrovolskaya O., Pont C., Sibout R., Martinek P., Badaeva E., Murat F., Chosson A., Watanabe N., Prat E., Gautier N., Gautier V., Poncet C., Orlov Y.L., Krasnikov A.A., Bergs H., Salina E., Laikova L., Salse J. FRIZZY PANICLE Drives Supernumerary Spikelets in Bread Wheat. Plant Physiol. 2015; 167(1):189-99. doi: 10.1104/pp.114.250043
      13. Doroshkov A.V., Konstantinov D.K., Afonnikov D.A., Gunbin K.V. The evolution of gene regulatory networks controlling Arabidopsis thaliana L. trichome development. BMC Plant Biol. 2019; 19(Suppl 1):53. doi: 10.1186/s12870-019-1640-2
      14. Fridborg I., Kuusk S., Moritz T., Sundberg E. The Arabidopsis dwarf mutant shi exhibits reduced gibberellin responses conferred by overexpression of a new putative zinc finger protein. Plant Cell. 1999; 11(6):1019-32. doi: 10.1105/tpc.11.6.1019
      15. Fullwood M.J., Liu M.H., Pan Y.F., Liu J., Xu H., Mohamed Y.B., Orlov Y.L., Velkov S., Ho A., Mei P.H., Chew E.G., Huang P.Y., Welboren W.J., Han Y., Ooi H.S., Ariyaratne P.N., Vega V.B., Luo Y., Tan P.Y., Choy P.Y., Wansa K.D., Zhao B., Lim K.S., Leow S.C., Yow J.S., Joseph R., Li H., Desai K.V., Thomsen J.S., Lee Y.K., Karuturi R.K., Herve T., Bourque G., Stunnenberg H.G., Ruan X., Cacheux-Rataboul V., Sung W.K., Liu E.T., Wei C.L., Cheung E., Ruan Y. An oestrogen-receptor-alpha-bound human chromatin interactome. Nature. 2009; 462(7269):58-64. doi: 10.1038/nature08497
      16. Goh W.S., Orlov Y., Li J., Clarke N.D. Blurring of high-resolution data shows that the effect of intrinsic nucleosome occupancy on transcription factor binding is mostly regional, not local. PLoS Comput Biol. 2010; 6(1):e1000649.
      17. Gorshkov V.M., Samarina L.S., Kulyan R.V., Malyarovskaya V.I., Ryndin A.V., Rakhmangulov R.S., Orlov Y.L. Challenges of in vitro conservation of Сitrus germplasm resources. 2019; 23(1):24-28 doi:10.18699/VJ19.457
      18. Grob S. Three-dimensional chromosome organization in flowering plants. Brief Funct Genomics. 2020; 19(2):83-91. doi: 10.1093/bfgp/elz024.
      19. Hao Q., Zhang L., Yang Y., Shan Z., Zhou X.A. Genome-Wide Analysis of the WOX Gene Family and Function Exploration of GmWOX18 in Soybean. Plants (Basel). 2019; 8(7):215. doi: 10.3390/plants8070215
      20. He X., Duque T.S., Sinha S. Evolutionary origins of transcription factor binding site clusters. Mol Biol Evol. 2012; 29(3):1059‐1070. doi:10.1093/molbev/msr277
      21. Hehl R., Norval L., Romanov A., Bülow L. Boosting AthaMap Database Content with Data from Protein Binding Microarrays. Plant Cell Physiol. 2016; 57(1):e4. doi: 10.1093/pcp/pcv156
      22. Ibarra I.L., Hollmann N.M., Klaus B., Augsten S., Velten B., Hennig J., Zaugg J.B. Mechanistic insights into transcription factor cooperativity and its impact on protein-phenotype interactions. Nat Commun. 2020; 11(1):124. doi:10.1038/s41467-019-13888-7
      23. Ignatieva E.V., Podkolodnaya O.A., Orlov Y.L., Vasiliev G.V., Kolchanov N.A. Regulatory genomics: combined experimental and computational approaches. Russian Journal of Genetics. 2015; 51(4): 334-352. doi: 10.1134/S1022795415040067
      24. Jeong H., Tombor B., Albert R., Oltvai Z.N., Barabási A.L. The large-scale organization of metabolic networks. Nature. 2000; 407(6804):651‐654. doi:10.1038/35036627
      25. Jin J., Zhang H., Kong L., Gao G., Luo J. PlantTFDB 3.0: a portal for the functional and evolutionary study of plant transcription factors. Nucleic Acids Res. 2014; 42(Database issue):D1182-7. doi: 10.1093/nar/gkt1016
      26. Kazemian M., Pham H., Wolfe S.A., Brodsky M.H., Sinha S. Widespread Evidence of Cooperative DNA Binding by Transcription Factors in Drosophila Development. Nuclei Acids Res. 2013; 41(17):8237-52. doi: 10.1093/nar/gkt598
      27. Khoueiry P., Girardot C., Ciglar L., Peng P.-C., Gustafson E.H., Sinha S., Furlong E.E. Uncoupling Evolutionary Changes in DNA Sequence, Transcription Factor Occupancy and Enhancer Activity. Elife 2017; 6:e28440. doi: 10.7554/eLife.28440
      28. Kolmykov S., Yevshin I., Kulyashov M., Sharipov R., Kondrakhin Y., Makeev V.J., Kulakovskiy I.V., Kel A., Kolpakov F. GTRD: an integrated view of transcription regulation. Nucleic Acids Res. 2021; 49(D1):D104-D111. doi: 10.1093/nar/gkaa1057
      29. Kulakova E.V., Spitsina A.M., Bogomolov A.G., Orlova N.G., Dergilev A.I., Chadaeva I.V., Babenko V.N., Orlov Y.L. Program for analysis of genome distribution of chromosome contacts in cell nucleus by the data obtained using ChIA-PET and Hi-C technologies, Program systems: Theory and applications, 2017, 8:1(32), pp. 219–242. (In Russian).
      30. Kulakova E.V., Spitsina A.M., Orlova N.G., Dergilev A.I., Svichkarev A.V., Safronova N.S., Chernykh N.S., Orlov Y.L. Supercomputer analysis of genomics and transcriptomics data revealed by high-throughput DNA sequencing. Program systems: theory and applications. 2015, 6:2(25):129–148. doi: 10.25209/2079-3316-2015-6-2-129-148
      31. Kulakovskiy I.V., Vorontsov I.E., Yevshin I.S., Sharipov R.N., Fedorova A.D., Rumynskiy E.I., Medvedeva Y.A., Magana-Mora A., Bajic V.B., Papatsenko D.A., Kolpakov F.A., Makeev V.J. HOCOMOCO: towards a complete collection of transcription factor binding models for human and mouse via large-scale ChIP-Seq analysis Nucleic Acids Res. 2018; 46(D1):D252-D259. doi: 10.1093/nar/gkx1106.
      32. Kuznetsov V.A., Orlov Y.L., Wei C.L., Ruan Y. Computational analysis and modeling of genome-scale avidity distribution of transcription factor binding sites in chip-pet experiments. Genome Inform. 2007; 19:83-94.
      33. Lai X., Daher H., Galien A., Hugouvieux V., Zubieta C. Structural Basis for Plant MADS Transcription Factor Oligomerization. Comput Struct Biotechnol J. 2019; 17:946‐953. doi:10.1016/j.csbj.2019.06.014
      34. Li G., Ruan X., Auerbach R.K., Sandhu K.S., Zheng M., Wang P., Poh H.M., Goh Y., Lim J., Zhang J., Sim H.S., Peh S.Q., Mulawadi F.H., Ong C.T., Orlov Y.L., Hong S., Zhang Z., Landt S., Raha D., Euskirchen G., Wei C.L., Ge W., Wang H., Davis C., Fisher-Aylor K.I., Mortazavi A., Gerstein M., Gingeras T., Wold B., Sun Y., Fullwood M.J., Cheung E., Liu E., Sung W.K., Snyder M., Ruan Y. Extensive promoter-centered chromatin interactions provide a topological basis for transcription regulation. Cell. 2012; 148(1-2):84-98. doi: 10.1016/j.cell.2011.12.014
      35. Liu F., Xu Y., Han G., Zhou L., Ali A., Zhu S., Li X. Molecular Evolution and Genetic Variation of G2-Like Transcription Factor Genes in Maize. PLoS One. 2016; 11(8):e0161763. 2016. doi:10.1371/journal.pone.0161763
      36. Lowry J.A., Atchley W.R. Molecular evolution of the GATA family of transcription factors: conservation within the DNA-binding Domain. J Mol Evol. 2000; 50(2): 103–115. doi: 10.1007/s002399910012
      37. Lu T., Dou Y., Zhang C. Fuzzy clustering of CPP family in plants with evolution and interaction analyses. BMC Bioinformatics. 2013; S10, 14. doi: 10.1186/1471-2105-14-S13-S10
      38. Muhammad I.I., Kong S.L., Akmar Abdullah S.N., Munusamy U. RNA-seq and ChIP-seq as Complementary Approaches for Comprehension of Plant Transcriptional Regulatory Mechanism. Int J Mol Sci. 2019; 21(1):167. doi: 10.3390/ijms21010167
      39. Narasimhan K., Pillay S., Huang Y.H., Huang Y.H., Jayabal S., Udayasuryan B., Veerapandian V., Kolatkar P., Cojocaru V., Pervushin K., Jauch R. DNA-mediated cooperativity facilitates the co-selection of cryptic enhancer sequences by SOX2 and PAX6 transcription factors. Nucleic Acids Res. 2015; 43(3):1513‐1528. doi:10.1093/nar/gku1390
      40. Naumenko F.M., Abnizova I.I., Beka N., Genaev M.A., Orlov Y.L. Novel read density distribution score shows possible aligner artefacts, when mapping a single chromosome. BMC Genomics. 2018; 19(Suppl 3): 92. doi: 10.1186/s12864-018-4475-6
      41. Orlov Y.L., Galieva A.G., Orlova N.G., Ivanova E.N., Mozyleva Y.A., Anashkina A.A. [Reconstruction of gene network associated with Parkinson disease for gene targets search]. Biomed Khim. 2021; 67(3):222-230. (in Russian) doi: 10.18097/PBMC20216703222
      42. Orlov Y.L., Bragin A.O., Babenko R.O., Dresvyannikova A.E., Kovalev S.S., Shaderkin I.A., Orlova N.G., Naumenko F.M. Integrated Computer Analysis of Genomic Sequencing Data Based on ICGenomics Tool In: Advances in Intelligent Systems, Computer Science and Digital Economics. Z. Hu et al. (Eds.): CSDEIS 2019, AISC 1127, International Journal of Intelligent Systems and Applications (IJISA) pp. 154–164, 2020. doi: 10.1007/978-3-030-39216-1_15
      43. Orlov Y.L., Salina E.A., Eslami G., Kochetov A.V. Plant biology research at BGRS-2018. BMC Plant Biol. 2019; 19 (Suppl 1):56. doi: 10.1186/s12870-019-1634-0
      44. Orlov Y.L., Thierry O., Bogomolov A.G., Tsukanov A.V., Kulakova E.V., Galieva E.R., Bragin A.O., Li G. [Computer methods of analysis of chromosome contacts in the cell nucleus based on sequencing technology data]. Biomed Khim. 2017; 63(5):418-422. doi: 10.18097/PBMC20176305418.
      45. Orlov Yu., Xu H., Afonnikov D., Lim B., Heng J.C., Yuan P., Chen M., Yan J., Clarke N., Orlova N., Huss M., Gunbin K., Podkolodnyy N., Ng H.H. Computer and statistical analysis of transcription factor binding and chromatin modifications by ChIP-seq data in embryonic stem cell J Integr Bioinform. 2012; 9(2): 211. doi: 10.2390/biecoll-jib-2012-211.
      46. Ramsey S.A. An Empirical Prior Improves Accuracy for Bayesian Estimation of Transcription Factor Binding Site Frequencies within Gene Promoters. Bioinform Biol Insights. 2016;9(Suppl 4):59-69. doi: 10.4137/BBI.S29330.
      47. Rolland T., Taşan M., Charloteaux B., Pevzner S.J., Zhong Q. A proteome-scale map of the human interactome network. Cell. 2014;159(5):1212‐1226. doi:10.1016/j.cell.2014.10.050
      48. Soltis D.E., Soltis P.S. The role of phylogenetics in comparative genetics. Plant Physiol. 2003; 132(4):1790-800. doi: 10.1104/pp.103.022509.
      49. Spitsina A.M., Bragin A.O., Dergilev A.I., Chadaeva I.V., Tverdokhleb N.N., Galiyeva E.R., Tabikhanova L.E., Orlov Y.L. Computer tools for analysis of transcriptomics data: program complex ExpGene, Program systems: Theory and applications, 2017, 8:2(33):45–68. (In Russian). doi: 10.25209/2079-3316-2017-8-2-45-68
      50. Tian F., Yang D.C., Meng Y.Q., Jin J., Gao G. PlantRegMap: charting functional regulatory maps in plants. Nucleic Acids Res. 2020; 48(D1): D1104-D1113. doi:10.1093/nar/gkz1020.
      51. Tong Q., Tsai J., Tan G., Dalgin G., Hotamisligil G.S. Interaction between GATA and the C/EBP family of transcription factors is critical in GATA-mediated suppression of adipocyte differentiation. Mol Cell Biol. 2005; 25(2):706-15. doi: 10.1128/MCB.25.2.706-715.200
      52. Tsukanov A.V., Orlova N.G., Dergilev A.I., Orlov Y.L. Programs for Statistical Analysis, Clusterization and Visualization of Genome Distribution of Transcription Factor Binding Sites. Vestnik NSU. Series: Information Technologies 2018; 16(3):51–63. (in Russian) doi: 10.25205/1818-7900-2018-16-3-51-63
      53. Villanueva J.M., Broadhvest J., Hauser B.A., Meister R.J., Schneitz K, Gasser C.S. INNER NO OUTER regulates abaxial- adaxial patterning in Arabidopsis ovules. Genes Dev. 1999; 13(23):3160‐3169. doi:10.1101/gad.13.23.3160
      54. Vityaev E.E., Orlov Yu.L., Vishnevsky O.V., Belenok A.S., Kolchanov N.A. Computer system "Gene Discovery" to search for patterns in eukaryotic regulatory nucleotide sequences. Molecular Biology. 2001; 35(6):810-817. doi: 10.1023/A:1013273932056
      55. Vityaev E.E., Orlov Yu.L., Vishnevsky O.V., Pozdnyakov M.A., Kolchanov N.A. Computer system "Gene Discovery" for promoter structure analysis. In Silico Biology 2002; 2(3):257-62.
      56. Wang J., Meng X., Dobrovolskaya O.B., Orlov Y.L., Chen M. Non-coding RNAs and Their Roles in Stress Response in Plants. Genomics Proteomics Bioinformatics. 2017; 15(5):301-312. doi: 10.1016/j.gpb.2017.01.007.
      57. Wasserman W.W., Sandelin A. Applied bioinformatics for the identification of regulatory elements. Nat Rev Genet. 2004; 5(4):276-87. doi: 10.1038/nrg1315
      58. Wheeler D.L., Smith-White B., Chetvernin V., Resenchuk S., Dombrowski S.M., Pechous S.W., Tatusova T., Ostell J. Plant genome resources at the national center for biotechnology information. Plant Physiol. 2005; 138(3):1280-8. doi: 10.1104/pp.104.058842.
      59. Winter C.M., Austin R.S., Blanvillain-Baufumé S., Reback M.A., Monniaux M., Wu M.F., Sang Y., Yamaguchi A., Yamaguchi N., Parker J.E., Parcy F., Jensen S.T., Li H., Wagner D. LEAFY target genes reveal floral regulatory logic, cis motifs, and a link to biotic stimulus response. Dev Cell. 2011; 19;20(4):430-43.
      60. Whyte W.A., Orlando D.A., Hnisz D., Abraham B.J., Lin C.Y., Kagey M.H., Rahl P.B., Lee T.I., Young R.A. Master transcription factors and mediator establish super-enhancers at key cell identity genes. Cell. 2013; 153(2):307-19. doi: 10.1016/j.cell.2013.03.035
      61. Zakhartsev M., Medvedeva I., Orlov Y., Akberdin I., Krebs O., Schulze W.X. Metabolic model of central carbon and energy metabolisms of growing Arabidopsis thaliana in relation to sucrose translocation. BMC Plant Biology. 2016; 16:262 doi: 10.1186/s12870-016-0868-3
      62. Zheng K., Ni Z., Qu Y., Ca Y., Yang Z., Sun G., Chen Q. Genome-wide identification and expression analyses of TCP transcription factor genes in Gossypium barbadense. Scientific Reports 2018; 8. 10.1038/s41598-018-32626-5
